# The impact of PSTD on service access among people who use drugs in Vancouver, Canada

**DOI:** 10.1186/s13011-021-00390-x

**Published:** 2021-06-26

**Authors:** Annemarie Goytan, William Lee, Huiru Dong, Kanna Hayashi, M. J. Milloy, Thomas Kerr

**Affiliations:** 1 British Columbia Centre on Substance Use, 400-1045 Howe Street, Vancouver, BC V6Z 2A9 Canada; 2 School of Population and Public Health, University of British Columbia, Vancouver, Canada; 3 Faculty of Health Sciences, Simon Fraser University, Burnaby, Canada; 4 Department of Medicine, University of British Columbia, Vancouver, Canada

**Keywords:** Post-traumatic stress disorder, Drug use, Mental health, Trauma, Healthcare, Service access

## Abstract

**Background:**

Settings throughout the United States and Canada are contending with high rates of drug-related overdose. This in turn has prompted efforts to more effectively engage people who use drugs (PWUD) in treatment and care. However, while co-morbid mental disorders are prevalent among PWUD and can undermine access to services, the impact of post-traumatic stress disorder (PTSD) on service access is not known. Therefore, we sought to assess whether PTSD is associated with difficulties accessing health and social services among PWUD in Vancouver, Canada.

**Methods:**

Survey data was derived from two prospective cohorts of PWUD in Vancouver, Canada for the period of April 2017 to November 2018. PTSD was assessed using the PTSD Checklist for the DSM-V (PCL-5). Generalized estimating equations (GEE) was used to estimate the relationship between PTSD and self-reported inability to access health and social services, after adjustment for confounders.

**Results:**

Among 810 participants included in our analysis, 316 (39.0%) participants qualified for a provisional PSTD diagnosis, and 117 (14.4%) reported difficulties accessing services. In a multivariable GEE analysis, a PTSD diagnosis (adjusted odds ratio = 1.69, 95% confidence interval: 1.12–2.55) was independently associated with difficulties accessing services.

**Conclusions:**

We found high rates of PTSD and self-reported difficulties accessing services among PWUD in Vancouver, as well as a positive association between PTSD and difficulties with service access. These findings highlight the need for trauma-informed approaches to service delivery for PWUD, as well as enhanced provider training specific to PTSD.

## Introduction

Settings throughout the United States and Canada continue to experience unprecedented levels of opioid overdoses driven largely by the increasing presence of synthetic fentanyl and related substances. This is true of Vancouver, British Columbia (BC), Canada, where in 2016 a public health emergency was declared in response to a rapid increase in overdose deaths; an 87% increase from 529 deaths in 2015 to 991 in 2016 [2]. Despite the existence of a range of harm reduction and treatment programs, estimated to have reduced total overdose fatalities from 1547 in 2018 to 983 in 2019, overdose rates remain high. The continued high overdose rate may be attributed in part to the stigma surrounding substance use, which creates a major barrier to accessing overdose prevention services and treatment programs [1, 22]. The crisis continues to worsen during the COVID-19 pandemic as 1068 overdose fatalities were recorded in the first 8 months of 2020 alone [2].

Comorbid mental disorders are prevalent among people who use drugs (PWUD) and are known to be associated with suboptimal health and social service access, poor treatment retention and outcomes, as well as elevated overdose risk [3, 9, 10, 20]. Among Canadians with a mental health disorder, those with a substance use disorder were over three times more likely than those without a substance use disorder to experience unmet mental health care needs [23]. Of particular concern is the high rate of post-traumatic stress disorder (PTSD) among PWUD. While the lifetime prevalence of PTSD in the general Canadian population is approximately 9.2% [24], the lifetime prevalence of PTSD among those with a substance use disorder is estimated to be between 26 and 52% [20]. PTSD is characterized by intrusions, avoidance behaviour, changes in mood and hypervigilance [8], all of which can impair goal-oriented thinking and behaviour, like pursuing supportive services and staying connected to services [17]. Additionally, recent data has indicated that a PTSD diagnosis almost doubles the overdose risk among people who use opioids [17]. Furthermore, many providers are untrained in trauma informed care (TIC), especially caring for PWUD [3], and can administer inappropriate care and perpetuate substance use related stigma, deterring PWUD from approaching services. While the ongoing overdose crisis has prompted efforts to improve access to health and social services and promote addiction treatment engagement [14], multiple factors can create service access barriers for PWUD with a PTSD diagnosis, heightening risks of poor treatment retention, outcomes and overdose death.

While access to services for PWUD with varying secondary mental health diagnoses like major depressive disorder (MDD) [3], anxiety disorder [15], and other psychiatric conditions [6] have been well investigated, the impact of PTSD on PWUD’s service access has not been well characterized. Therefore, we sought to explore the relationship between PTSD and difficulties accessing health and social services among PWUD in Vancouver.

## Methods

Data were derived and combined from two ongoing and harmonized prospective cohort studies of PWUD in Vancouver, Canada: the Vancouver Injection Drug Users Study (VIDUS) and the AIDS Care Cohort to evaluate Exposure to Survival Services (ACCESS). Both cohorts have been recruited via snowball sampling, self-referral and street outreach since 1996 and have been described in detail elsewhere [21, 27]. In short, VIDUS eligible participants are HIV-negative individuals who have injected illicit drugs at least once in the month prior to enrolment. ACCESS eligible participants are HIV-positive individuals who used illicit drugs other than or in addition to cannabis in the month prior to enrolment. At baseline and semiannually, both VIDUS and ACCESS participants complete a harmonized  interviewer-administered questionnaire which gathers quantitative information on demographic characteristics, substance use and risk behaviours, drug use treatment and use of health services information. Participants from each cohort also provide a venous blood sample for serologic testing. All participants provide informed consent and are given a $40 (CAD) stipend after each study visit. The use of VIDUS and ACCESS data in the current study have been approved by the University of British Columbia’s Research Ethics Board.

Our analyses are restricted to VIDUS and ACCESS participants who completed at least two study interviews at two distinct time points between April 2017 and November 2018. The primary outcome of interest was the inability to access services, assessed by the question: “*in the last 6 months, was there a time you were in need of a service (e.g., housing, counselling, police) but could not obtain it*?”. This definition has been used in previous studies [19, 25] to assess service access barriers among PWUD and was coded dichotomously (yes vs. no).

To assess for PTSD, each participant completed the PTSD Checklist for DSM-5 (PCL-5). This 20-item questionnaire evaluates PTSD symptoms experienced in the previous month. Each item is answered on a five-point scale (zero = not at all to four = extremely), and scores range from 0 to 80. Consistent with previous studies, a provisional PTSD diagnosis was defined as a PCL-5 score of 31 or higher [26].

After reviewing the literature [3, 17, 19], other characteristics considered as potential confounders included age (≥median vs. <median), sex (male vs. non-male), ethnicity (white vs. Black Indigenous or other People of Colour [BIPOC]), and within the last 6 months prior to data collection interviews: homelessness (yes vs. no), incarceration (yes vs. no), sex work (yes vs. no), overdose (yes vs. no), employment (yes vs. no), alcohol binge (yes vs. no), daily use of prescription opioids (yes vs. no), heroin (yes vs. no), crack (yes vs. no), cocaine (yes vs. no), and methamphetamine use (yes vs. no).

Our study aims to assess whether trauma, operationalized by a provisional PTSD diagnosis, is associated with the inability to access services for PWUD. Since repeated measures were available for each participant, we applied generalized estimating equation (GEE) logistic regression with logit-link function for the analysis of correlated data. By using an exchangeable correlation structure that adjusts for multiple observations for each participant, this method identified factors associated with the outcome across the entire study period. As a first step, a bivariable GEE analysis was used to determine factors associated with inability to access services in the last 6 months. In this analysis, the explanatory variables significantly associated with the outcome were PTSD, homelessness, recent incarceration, overdose, daily crack use and daily methamphetamine use. To fit the multivariable model, we began with all explanatory variables found to be associated with the outcome at *p* < 0.10 in bivariable analyses, then generated a series of reduced models by removing each secondary explanatory variable one at a time. For each of these models we assessed the relative change in the coefficient for PTSD. The secondary explanatory variable that resulted in the smallest absolute relative change in the coefficient for PTSD was then removed. Secondary variables continued to be removed through this process until the smallest relative change exceeded 5%. Remaining variables were considered confounders and were included in the final multivariable model. Data analyses were conducted using SAS version 9.4 (SAS Institute, Cary, NC, USA) and all *p*-values were two-sided.

## Results

In total, 810 individuals were included in this analysis, including: 488 (60.3%) males, 289 (35.7%) females, 10 (1.2%) transwomen, and 2 (0.4%) two-spirited individuals. Also included were: 343 (42.3%) white individuals, 338 (41.7%) individuals of Indigenous ancestry, 111 (13.7%) who identified as being a person of colour other than black, and 13 (1.6%) black individuals. Further, 645 (79.6%) of participants identified themselves as being “straight”, 45 (5.6%) identified as being gay, 4 (0.5%) identified as being lesbian, 3 (0.4%) identified as being bisexual, and 3 (0.4%) identified as being two-spirited, and 9 (1.1%) identified as belonging to another unspecified sexual minority group.

The median age at baseline was 50.5 years [interquartile range = 41.8–56.1]. Overall, 117 (14.4%) of participants reported needing a service but were unable to access it during the study period, and 316 (39.0%) participants qualified for a provisional PTSD diagnosis.

In bivariable GEE analyses (as shown in Table 1), factors positively associated with the inability to access services were PTSD, homelessness, recent incarceration, overdose, daily crack use and daily methamphetamine use, while older age was negatively associated with an inability to access services (all *p* < 0.05). In a multivariable GEE analysis (as shown in Table 1), PTSD (adjusted odds ratio [AOR] = 1.69, 95% confidence interval [CI]: 1.12–2.55; *p* = 0.007) remained independently associated with an inability to access service, as did homelessness (AOR = 13.84, 95% CI: 8.96–21.36; *p* < 0.001).

**Table 1:** Bivariable and multivariable GEE analysis of factors associated with inability to access services among PWUD

	Unadjusted		Adjusted	
Characteristic	Odds Ratio (95% CI)	***p -*** value	Odds Ratio (95% CI)	***p -*** value
PTSD				
(≥31 vs. < 31)	2.13 (1.44–3.15)	< 0.001*	1.69 (1.12–2.55)	< 0.0012*
Median Age				
(≥median vs. <median)	0.45 (0.30–0.67)	< 0.001*	–	–
Gender				
(female vs. male)	1.01 (0.66–1.53)	0.971	–	–
(gender minority vs. male)	1.61 (0.45–5.72)	0.464	–	–
Sexual Orientation				
(straight vs. others)	0.62 (0.38–1.01)	0.055	0.55 (0.33–0.92)	0.022*
Ethnicity				
(white vs. BIPOC)*	1.10 (0.74–1.63)	0.636	–	–
Homelessness^a^				
(yes vs. no)	13.66 (8.96–20.83)	< 0.001*	13.84 (8.96–21.36)	< 0.001*
Recent Incarceration^a^				
(yes vs. no)	5.06 (2.66–9.62)	< 0.001*	–	–
Sex Work Involvement^a^				
(yes vs. no)	1.59 (0.88–2.86)	0.124	–	–
Overdose^a^				
(yes vs. no)	2.51 (1.59–3.98)	< 0.001*	–	–
Employment^a^				
(yes vs. no)	0.95 (0.62–1.45)	0.815	–	–
Alcohol Binge^a^				
(yes vs. no)	1.50 (0.94–2.40)	0.090	–	–
Daily Prescription Opioid Use^a^				
(yes vs. no)	0.85 (0.24–2.97)	0.800	–	–
Daily Heroin Use^a^				
(yes vs. no)	1.87 (1.26–2.76)	0.002*	–	–
Daily Crack Use^a^				
(yes vs. no)	0.54 (0.27–1.10)	0.088	–	–
Daily Cocaine Use^a^				
(yes vs. no)	0.70 (0.27–1.85)	0.478	–	–
Daily Meth Use^a^				
(yes vs. no)	2.09 (1.33–3.29)	0.001*	–	–
Cohort				
(ACCESS vs. VIDUS)	0.58 (0.39–0.88)	0.010*		

^a^ Behaviours refer to activities in the last 6 months

*BIPOC refers to Black, Indigenous or other People of Colour

## Discussion

Our study found that a significant proportion of participants self-reported difficulties accessing services, and over a third met the provisional PTSD diagnosis. In our multivariable analysis, inability to access services among PWUD was positively associated with a provisional PTSD diagnosis.

One explanation for such findings is that PTSD can impair cognition, negatively affecting the ability to obtain services [17]. Therefore, impaired goal-oriented thinking coupled with the need for relief from emotional pain [7, 17] can prompt PWUD to seek immediate self-soothing resources (e.g., use of opioids), rather than pursuing supportive services and staying connected in services, which often require long wait lists and specific criteria [4]. Similar to the current study, Beaulieu et al. [3] found that amongst VIDUS and ACCESS participants, major depressive disorder (MDD) diagnosis among PWUD was positively and independently associated with reported barriers to accessing health care. A possible explanation for this relationship proposed by the authors was service provider displays of stigma. As the current study involved the same cohorts as that of Beaulieu et al. [3], the same explanation may extend to the current study. Displays of stigma, related to mental illness, substance use and homelessness, may deter clients from reaching out for services or staying in touch with services, despite needing such supports. As well, as suggested by Beaulieu et al. [3], service providers may be unfamiliar with how to assist PWUD who are diagnosed with PTSD, and their lack of confidence in serving this population results in inconsistent support and compassion fatigue. Further, avoidance of addressing difficult issues, like trauma, may be interpreted as mistreatment or stigmatized views by clients, which may further deter PWUD from reaching out or staying connected with much needed supports and services. Thus, critical next steps involve further training and education for service providers on the issues of drug use and trauma related mental illness, allowing them to better respond to and work with clients from backgrounds of trauma [11]. A study by Morrissey et al. [18] found that 31% of female PWUD with severe PTSD symptoms given TIC for 12 months had their trauma symptoms reduced to moderate or less, compared to only 16% given non-TIC. Furthermore, clients who receive TIC report feeling safer and better able to self-advocate in service settings [13], making clients more likely to access and remain connected to services provided in a TIC context. Such changes have potential to create a more suitable emotional climate to better serve PWUD facing these challenges, with the broader aim of reducing perceived barriers to accessing the support that they need.

Additionally, past reviews suggest that PWUD commonly report an inability to access community mental health services like counselling [7], consistent with the current study. This could be due to the lack of funding for and scarcity of such services, or could be attributed to the limited amount of services offered by providers, which was highlighted as a primary barrier to service access in a study involving PWUD in Edmonton, Canada [12]. Those seeking services may have been able to gain initial access; however, they were not granted the appropriate amount of services to meet their needs, whether it be frequency or duration of services, therefore creating perceived unmet needs in accessing adequate services. This indicates a need to expand community mental health services provided to accommodate clients with higher service needs, as PWUD living with PTSD generally need mental health care more frequently and longer term than those in the general population [15].

The current study is limited by the fact that participants in our study were not randomly selected, which limits the generalizability of our results to those close in characteristic to the study sample. Further, we relied on self-reported data for both measures of the primary outcome and predictor variable, and as such, these data are subject to response biases like social desirability and recall biases [5, 16]. Our data was also observational, therefore no causal relationship between variables can be drawn. Finally, though we did adjust for potential confounders in our analyses, additional unmeasured factors may have affected the results.

## Conclusion

Overall, we found that PWUD are more likely to experience the inability to access services if they are experiencing PTSD. Our study suggests that service providers may benefit from education and training in TIC approaches to enhance access for PWUD with PTSD, as this client population might have difficulties navigating the system due to the mental and emotional difficulties that accompany PTSD. At a broader level, efforts should be made to expand services to account for more complex client needs and should accommodate those with concurrent mental health disorders who have higher service needs.

## Data Availability

The datasets used and/or analysed during the current study are available from the corresponding author on reasonable request.

## Abbreviations

PTSD: Post-traumatic stress disorder
PWUD: People who use drugs
TIC: Trauma informed care
PCL-5: PTSD Checklist for the DSM-V
VIDUS: Vancouver Injection Drug Users Study
ACCESS: AIDS Care Cohort to evaluate Exposure to Survival Services
MDD: Major depressive disorder
GEE: Generalized estimating equation
